# Identification, Molecular Docking Mechanism and Cellular Activity of Selenium-Enriched ACE Inhibitory Peptides from Oysters

**DOI:** 10.3390/molecules30244818

**Published:** 2025-12-18

**Authors:** Zhuangzhuang Yue, Zhen Xia, Fei Xu, Bingbing Chen, Shufei Jiao, Xingtang Liang, Yanzhen Yin, Jianyin Miao

**Affiliations:** 1 Guangxi Key Laboratory of Green Chemical Materials and Safety Technology, School of Petroleum and Chemical Engineering, Beibu Gulf University, Qinzhou 535011, China; yzz16987@163.com (Z.Y.); 195577049198@163.com (F.X.); jiaoshufei2013@163.com (S.J.); yinyanzhen2018@163.com (Y.Y.); 2 Guangdong Provincial Key Laboratory of Nutraceuticals and Functional Foods, College of Food Science, South China Agricultural University, Guangzhou 510642, China; 15734900070@163.com (Z.X.); 18177786164@163.com (B.C.)

**Keywords:** Se-enriched ACE inhibitory peptides, molecular docking, oyster protein, antihypertensive activity

## Abstract

Selenium-enriched oyster proteins were hydrolyzed using trypsin to obtain peptides with angiotensin-I-converting enzyme (ACE) inhibitory activity. The hydrolysate was purified by ultrafiltration and two-step reversed-phase high-performance liquid chromatography (RP-HPLC), yielding the most active fraction M4-2 (selenium content: 37.00 ± 0.56 mg/kg; IC_50_: 0.774 mg/mL, significantly lower than the IC_50_ of the crude hydrolysate, 2.801 mg/mL). This fraction was further analyzed by LC-MS/MS and molecular docking, leading to the identification of 91 selenium-containing peptide sequences. Two novel peptides, SeMFRTSSK and QASeMNEATGGK, showing strong binding affinities (−9.8 and −9.0 kcal/mol, respectively), were selected. Molecular docking revealed that SeMFRTSSK bound to key residues in the ACE active pocket via hydrogen bonds, whereas QASeMNEATGGK interacted with the Zn^2+^ active center. Cellular assays using EA.hy926 cells demonstrated that both peptides were non-cytotoxic at concentrations up to 0.25 mg/mL. At 0.025 mg/mL, SeMFRTSSK and QASeMNEATGGK enhanced cellular NO release by 202.65% and 273.45%, respectively, while suppressing Endothelin-1 (ET-1) secretion by 18.03% and 27.86%, compared to the blank control group. Notably, these peptides induced higher levels of NO release and greater suppression of ET-1 secretion than those in the captopril-treated positive control group. These findings support selenium-enriched oyster-derived peptides as potential natural antihypertensive ingredients.

## 1. Introduction

Hypertension is a typical chronic disease that can trigger a series of complications, such as stroke and heart failure, and is increasingly afflicting younger populations [1]. Inhibiting Angiotensin I-converting enzyme (ACE) is one of the effective strategies for treating hypertension due to its core functions in the renin-angiotensin system (RAS) and the kallikrein-kinin system (KKS) [2]. The commercial chemosynthetic ACE inhibitors, including captopril, enalapril, and benazepril, exhibit significant therapeutic efficacy for hypertension. However, they also pose risks of adverse reactions, such as renal function impairment, dry cough, and skin rashes [1]. ACE inhibitory peptides are regarded as one of the preferable substitutes for chemosynthetic ACE inhibitors on account of their enhanced safety and superior absorbability, thus attracting significant attention [3]. Food-derived ACE inhibitory peptides, owing to their extensive sources, have been extracted from diverse protein materials like cheese [4], fish [5], beans [1,6], and nuts [7]. Beyond ACE inhibition, marine-derived bioactive peptides also exhibited other health-promoting effects, such as antioxidant activity, anti-fatigue, and blood glucose-lowering activities. For instance, a recent study on umami peptides derived from tuna skeletal myosin revealed that LADW and EEAEGT could inhibit α-glucosidase and starch hydrolysis, highlighting their potential in managing postprandial hyperglycemia [8].

The oyster is one of the most widely cultured shellfish globally (with a production exceeding 6.2 million tons in 2019), and more than 80% of oysters are produced in China [9]. Nevertheless, most oysters are consumed directly, and deep-processing products of oyster with high nutritional and medicinal values are still lacking [10]. The oyster is a well-known medicinal food in traditional Chinese medicine. Due to its high protein content (reaching up to 45–57% of the dry weight), oyster emerges as a high-quality candidate for preparing bioactive peptides [11]. Numerous studies have confirmed the functional properties of oyster protein peptides, including antioxidant [12], antihypertensive [13], anti-fatigue [14], immune regulation [15], and anti-tumor effects [16]. Furthermore, oysters are rich in trace elements such as zinc (Zn) and selenium (Se), with the selenium concentration tested to be 0.36–1.30 mg/kg [17]. Because of its crucial role in preventing cardio-vascular and cerebrovascular diseases, Se is considered an indispensable trace element for humans [18]. In recent years, organic-Se has drawn extensive attentions due to its advantages of higher bioactivity, safety, and bioavailability compared to inorganic-Se [19]. Generally, inorganic Se can be converted into the organic form by combining with poly-saccharides, peptides, and proteins in different organisms [20]. Se-enriched peptides, an important category of organic Se, which possess both peptide and Se functions, have become a hotspot in the field of bioactive peptides [21]. Liu et al. demonstrated that the selenium in the Se-enriched brown rice protein antioxidant peptide (SeMet-Pro-Ser) exhibited a synergistic effect on the antioxidant activity of the peptides [22]. Zhu et al. isolated two novel ACE inhibitory peptides, namely Se-TAPep 1 and TAPep 1, from selenium-enriched and selenium-free tea alkali-soluble proteins, respectively [23]. They showed that the ACE inhibitory activity of Se-TAPep 1 was significantly superior to that of TAPep 1 [23]. However, most current studies on Se-enriched ACE inhibitory peptides highlight the preparation process and purification of selenium-enriched peptides. The structure and specific active mechanism of Se-enriched peptides, especially those derived from marine animals, remain unknown.

In this study, Se-enriched oysters from the Beibu Gulf coast in Guangxi, China, were utilized as a raw material to investigate their potential as a valuable source of ACE inhibitory peptides. Firstly, the enzymatic preparation, purification, and identification of amino acid sequences for ACE inhibitory peptides derived from Se-enriched oysters were investigated. Subsequently, the potential ACE inhibitory peptides were screened and obtained through molecular docking technology. The binding pattern and active sites of these peptides toward ACE were further analyzed using molecular docking to reveal the antihyper-tensive mechanism. Finally, the ACE inhibitory activities of the corresponding synthetic Se-enriched peptides in cells were studied. This work provides a new approach for the high-value utilization of oysters and a theoretical basis for the development of Se-enriched bioactive peptides with antihypertensive activity.

## 2. Results and Discussion

### 2.1. ACE Inhibitory Activities of Se-Enriched Oyster Hydrolysates Derived from Various Proteases

**Figure 1:**
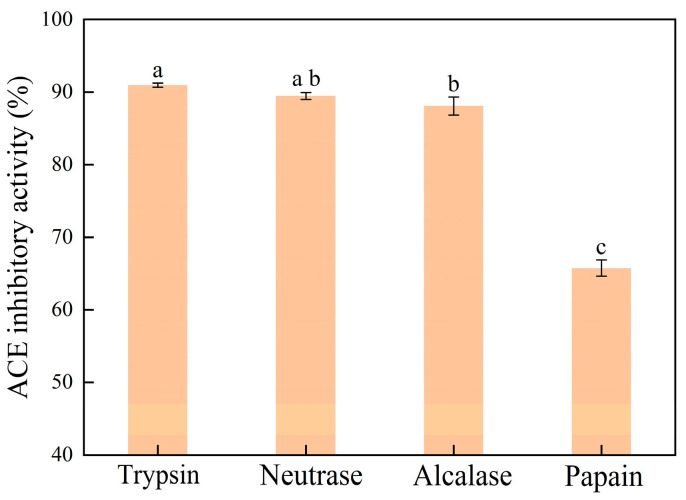
ACE inhibitory activities for Se-enriched oyster hydrolysates prepared by different proteases. Bars with different lowercase letters indicate significant differences (*p* < 0.05) as determined by Tukey’s test.

The Se-enriched oyster was hydrolyzed using four representative proteases, i.e., alcalase, neutrase, papain, and trypsin. The ACE inhibitory activities of the resultant hydrolysates were measured. As shown in Figure 1, the Se-enriched oyster-derived hydrolysates exhibited different ACE inhibitory activities depending on the type of protease used, indicating the substrate specificity of the proteases. The hydrolysate derived from the trypsin-triggered process exhibited the highest ACE inhibitory activity, reaching 90.95 ± 0.28%. In contrast, the papain-induced hydrolysate presented a minimum ACE inhibitory activity of 65.76 ± 1.13%. Due to the high ACE inhibitory activity of its product, trypsin was selected for the large-scale preparation of Se-enriched oyster hydrolysate.

### 2.2. Isolation, Purification, and ACE Inhibitory Activity of Se-Enriched Oyster Peptide

**Figure 2:**
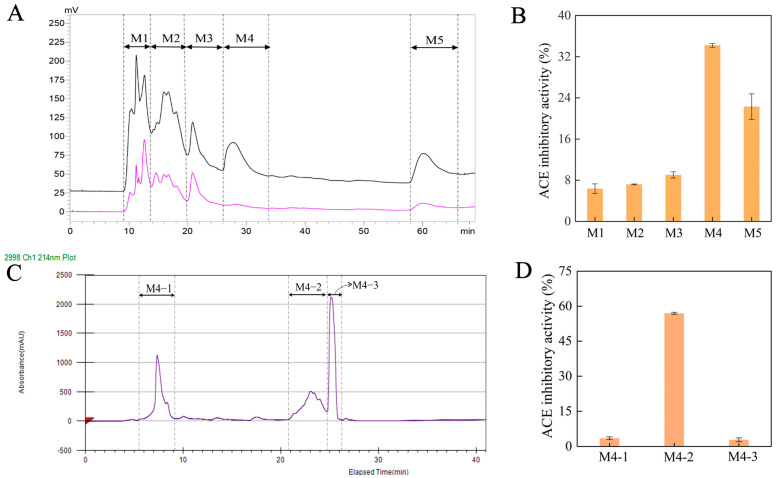
(**A**) Chromatogram profiles for the ultrafiltration fraction (MW < 10 kDa) of Se-enriched oyster hydrolysate acquired by preparative RP-HPLC at the absorption wavelengths of 214 nm (pink curve) and 280 nm (black curve); (**B**) the ACE inhibitory activity for the fractions of M1, M2, M3, M4 and M5 estimated at 1.0 mg/mL; (**C**) chromatogram profiles for the M4 fraction by preparative RP-HPLC; (**D**) the ACE inhibitory activity for the fractions of M4-1, M4-2 and M4-3 measured at 1.0 mg/mL.

Numerous studies have verified that peptides with low molecular weight typically exhibit superior ACE inhibitory activity compared to those with high molecular weight [24,25]. Herein, the Se-enriched oyster hydrolysates with a small molecular weight (<10 kDa) were selected for further purification. These hydrolysates were purified using preparative RP-HPLC based on their molecular weights, yielding five major fractions. These fractions were collected and designated as M1, M2, M3, M4, and M5, respectively (Figure 2A). The ACE inhibitory activities of these fractions were then evaluated at the concentration of 1.0 mg/mL. As shown in Figure 2B, the M4 fraction presented significantly the highest ACE inhibitory activity than the other fractions (*p* < 0.05). The M4 fraction was thus used for further separation on the SunFire Prep C18 column and revealed three fractions, namely M4-1, M4-2, and M4-3, respectively (Figure 2C). At the concentration of 1.0 mg/mL, M4-2 indicated a dramatically higher ACE inhibitory activity than M4-1 and M4-3 (Figure 2D). Both M4-1 and M4-3 featured a poor ACE inhibitory activity of <3.5% (*p* < 0.05). In addition, the ACE inhibitory activity of the subfraction M4-2 was substantially superior to that of its parent fraction, M4 (56.96 ± 0.11% vs. 34.20 ± 0.41%), evidencing the main contribution of M4-2 to the ACE inhibitory activity. Therefore, M4-2 was collected and employed for further analysis.

### 2.3. The Change in Se Content and IC_50_ During the Peptide Preparation

In order to track the selenium content of the samples during peptide preparation, we measured the Se content of the M4, M4-2 fraction, and the oyster powder. As listed in Table 1, the trypsin-triggered hydrolysate revealed a Se content of 2.44 ± 0.71 mg/kg, which is greater than that of the raw material (1.30 ± 0.07 mg/kg). Impressively, the M4-2 fraction highlighted a Se content of 37.00 ± 0.56 mg/kg, which is 15 times higher than that of the trypsin-derived hydrolysate. This result indicated that Se was well enriched during the separation and purification process. This result indicated that Se was well enriched during the separation and purification process. This enrichment can be explained by the selective separation and concentration based on the hydrophobicity of selenomethionine (SeMet). SeMet is more hydrophobic than methionine, which confers greater hydrophobicity to peptides containing it. Consequently, during reverse-phase high-performance liquid chromatography (RP-HPLC), SeMet-containing peptides exhibited stronger retention on the hydrophobic stationary phase and were eluted within a specific window (fraction M4-2). By specifically collecting this fraction, we effectively enriched peptides that originally contained SeMet from the complex hydrolysate, rather than creating new selenium species. Therefore, this process represents a selective purification and concentration based on the inherent physicochemical properties of the target peptides. The IC_50_, a key indicator for the capacity of a peptide toward ACE inhibition, represents the concentration of ACE inhibitory peptide at which its ACE inhibitory activity results in a 50% decrease. In general, a lower IC_50_ value implies a higher ACE inhibitory capacity of the peptide [26]. As expected, the M4-2 fraction featured an IC_50_ of 0.774 mg/mL, which is significantly lower than that of the trypsin-derived hydrolysate (IC_50_ = 2.801 mg/mL). Taken together, the successful enrichment of SeMet-containing peptides through hydrophobic interaction, coupled with the significantly enhanced ACE inhibitory activity of the M4-2 fraction, strongly suggests that the incorporated selenium (as SeMet) plays an important role in the bioactivity of the oyster-derived peptides. This strong correlation, combined with the molecular docking predictions detailed in Section 2.5 which suggest potential involvement of SeMet in key interactions, indicates that selenium (in the form of SeMet) may contribute positively to the bioactivity of these oyster-derived peptides. The preparative HPLC chromatographic profiles and mass spectrometric characterization data for the synthetic peptides SeMFRTSSK and QASeMNEATGGK are provided in Supplementary Materials Figures S1–S4.

### 2.4. Identification of ACE Inhibitory Peptides and Screening of Peptide with Highly Active

In this study, the abbreviation SeM denotes the residue of selenomethionine, and SeC denotes a putative selenium for sulfur substitution on a cysteine residue. To identify the potential ACE inhibitory peptides, the peptide composition and amino acid sequence of the active fraction M4-2 were analyzed by LC-MS/MS. As presented in Table 2, 91 Se-enriched peptide sequences with an average local confidence ALC ≥ 60% were identified. The complete list of these peptides, grouped by the physicochemical property of their N-terminal residue and sorted by binding affinity within each group, is provided in Supplementary Materials Table S1. These peptide sequences, with a molecular weights ranging from 539.1606 to 1211.4475 Da, contained 4 to 13 amino acid residues, indicating the dominance of short peptides.

Molecular docking is an intuitive and effective tool for simulating the structure-activity relationships between receptors and ligands [27]. The model results of molecular docking are expressed as binding energy. Generally, a lower binding energy value signifies a higher stability of the ligand-receptor complex [28]. Herein, the potential ACE inhibitory peptides were screened using molecular docking technology. As listed in Table 2, the binding affinity of the peptides to ACE (PBD ID: 1O8A) ranged from −9.8 to −6.7 kcal/mol. Among the studied peptides, SeMFRTSSK (903.3717 Da) and QASeMNEATGGK (1053.3994 Da) emerged as the two peptides with the highest binding affinity to ACE. Their binding energies were −9.8 kcal/mol and −9.0 kcal/mol, respectively. In general, ACE inhibitory peptides with a short chain of 2 to 12 amino acid residues exhibit high activity, while long chain peptides show low activity due to poor binding to the active site of ACE [29]. Additionally, positively charged amino acid residues (such as Lys, Arg, His) and hydrophobic amino acid residues (e.g., Leu, Pro, Val, Phe, Met, Trp and Ile) reveal positive effects on ACE inhibitory activity [18]. Moreover, previous studies have suggested that a hydrophobic amino acid residue as the N-terminal is beneficial for the activity of ACE inhibitory peptides [30]. In this work, SeMFRTSSK and QASeMNEATGGK are short chain peptides composed of 7 and 10 amino acid residues, respectively, and the Se in SeMFRTSSK and QASeMNEATGGK exists in the form of SeMet. This structure corresponds to the typical structure of high-activity ACE inhibitory peptides [30]. Therefore, SeMFRTSSK and QASeMNEATGGK with potential ACE inhibitory activity were synthesized for subsequent analysis.

To evaluate the potency of the selenium-enriched oyster peptides (SeMFRTSSK and QASeMNEATGGK) identified in this study, we compared them with other selenium-containing peptides reported in the literature. For example, the tripeptide Se-MPS derived from selenized brown rice protein has an IC_50_ of 0.087 mg/mL [22], while the selenium-enriched tea protein peptide Se-TAPep 1 exhibits an IC_50_ of 1.29 mM [23]. In comparison, based on the IC_50_ of the M4-2 fraction (0.774 mg/mL) and the molecular weights of the two synthetic peptides (SeMFRTSSK: 903.37 Da; QASeMNEATGGK: 1053.40 Da), their estimated molar IC_50_ values are approximately 0.857 mM and 0.735 mM, respectively. Although their activity is not the strongest, it still falls within the effective range observed for food-derived ACE inhibitory peptides, indicating that oyster-derived selenium-containing peptides are a promising source of antihypertensive agents, with potency comparable to other selenium-enriched protein hydrolysates.

### 2.5. Molecular Docking Analysis of the Interaction Between Se-Enriched Peptides and ACE

**Figure 3:**
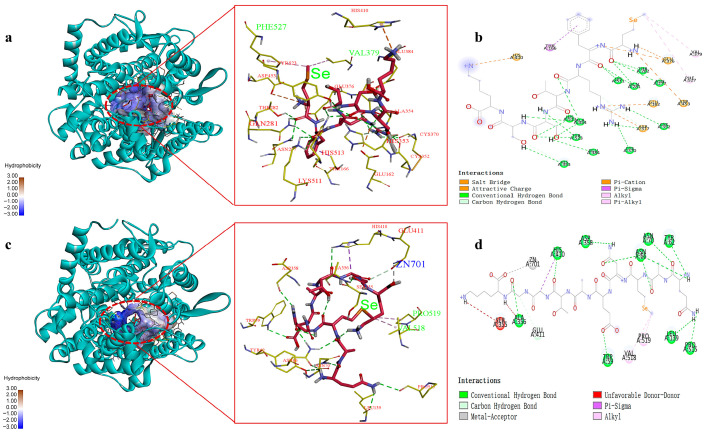
Molecular docking results for SeMFRTSSK and QASeMNEATGGK binding with ACE molecule. (**a**) 3D details of ACE and SeMFRTSSK interactions; (**b**) 2D diagram showing interactions between SeMFRTSSK and ACE amino acid residues; (**c**) 3D details of ACE and QASeMNEATGGK interactions; (**d**) 2D diagram showing interactions between QASeMNEATGGK and ACE amino acid residues.

In order to explore the ACE inhibitory mechanisms, molecular docking technology was further employed to analyze the binding mode and action sites of SeMFRTSSK and QASeMNEATGGK with respect to the receptor ACE. Typically, ACE has three major active site regions, i.e., S1, S2, and S1’. The S1 contains amino acid residues Ala354, Glu384, and Tyr523, forming an active pocket; the S2 pocket is composed of amino acid residues Gln281, His353, Lys511, His513, and Tyr520, while the S1’ active pocket possesses residue Glu162 [27]. In addition, Zn^2+^ can coordinate with amino acid residues His383, His387, and Glu411 to form another active site [31]. Moreover, interactions such as hydrogen bonding and hydrophobic interaction can affect the stability and inhibitory activity of the complexes of ACE and inhibitor [26]. The simulation results, including the 3D and 2D diagrams for the interactions between ACE and Se-containing peptides, SeMFRTSSK and QASeMNEATGGK, are presented in Figure 3.

As shown in Figure 3a,c, the optimal docking conformations of ACE and peptides SeMFRTSSK and QASeMNEATGGK support the formation of stable complexes. The corresponding interactions and active sites are tabulated in Table 3.

The main interaction modes between SeMFRTSSK and ACE include hydrogen bonds, hydrophobic interaction, and electrostatic interaction, while the complex of QASeMNEATGGK and ACE is mainly formed through hydrogen bonds, hydrophobic interaction, and metal-receptor interaction. For SeMFRTSSK, it can bind to the amino acid residues in the S1, S2, and S1’ active pockets of ACE, forming a highly stable complex [32]. Specifically, SeMFRTSSK forms hydrogen bonds with the Ala354, Glu384, Tyr523, Gln281, His353, Lys511, and His513 amino acid residues in the ACE active pocket. These hydrogen bond connections are similar to the connections between ACE and captopril, a representative antihypertensive drug. Captopril interacts with Gln281, His353, Lys511, and His513 amino acid residues of ACE through hydrogen bonds. This result evidences the high efficacy of SeMFRTSSK in ACE inhibition. For QASeMNEATGGK, it connects to several sites of ACE, including Trp62, Asn66, Leu139, Ala356, His410, Glu411, and Pro515 amino acid residues via hydrogen bonds. Among these sites, the binding to Glu411, as suggested by the docking pose, may potentially distort the tetrahedral coordination geometry of Zn^2+^, which could impair the catalytic activity of ACE [33]. Meanwhile, the formation of the metal-receptor interaction between QASeMNEATGGK and the active center Zn701 of ACE could hinder the combination of ACE and Zn^2+^, which might thereby suppress the catalytic activity of ACE [34]. These docking results suggest possible interactions with Glu411 and Zn^2+^ that may influence the catalytic geometry, although experimental validation is required.

Furthermore, the SeMet in both SeMFRTSSK and QASeMNEATGGK played an important role in the combination of ACE and peptides, which could eliminate the catalytic activity of ACE. For instance, the SeMet of SeMFRTSSK connected to the amino acid residues Val379 and Phe527 of ACE through hydrophobic interaction. Similarly, the hydrophobic interactions between QASeMNEATGGK and ACE were also established by the SeMet and the amino acid residues Val518 and Pro519. However, further research is required to explore the specific role of Se in ACE inhibition.

### 2.6. Cellular Antihypertensive Activity of Se-Enriched Oyster-Derived ACE Inhibitory Peptides

#### 2.6.1. Effect of Se-Enriched Oyster-Derived ACE Inhibitory Peptides on the Viability of EA.hy926 Cells

**Figure 4:**
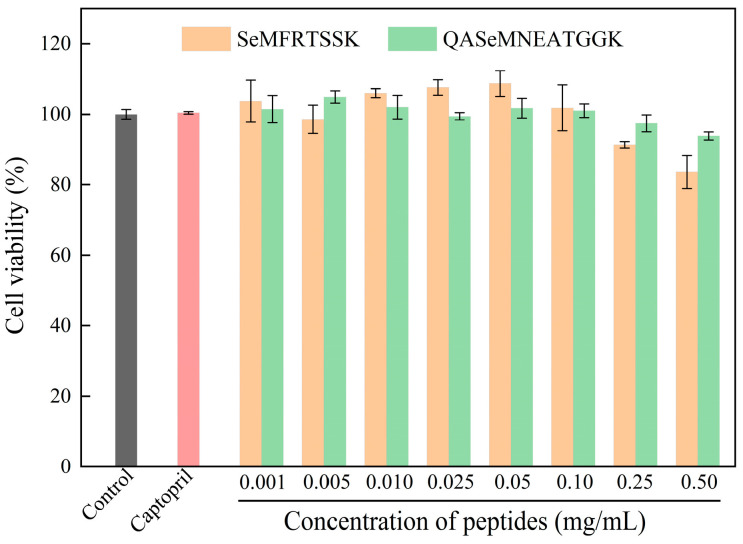
Effect of oyster-derived Se-enriched ACE inhibitory peptides on the cell viability of EA.hy926 cells.

The effects of SeMFRTSSK and QASeMNEATGGK on the viability of EA.hy926 cells were determined by the MTT assay. As depicted in Figure 4, at concentrations ranging from 0.001 to 0.25 mg/mL, cells treated with SeMFRTSSK exhibited a viability of greater than 91%, indicating very low cytotoxicity. Impressively, SeMFRTSSK induced cell proliferation when the concentration was between 0.01 mg/mL and 0.05 mg/mL. For QASeMNEATGGK, the treated cells showed a viability of greater than 93% at a concentration range of 0.001 to 0.50 mg/mL. In conclusion, SeMFRTSSK and QASeMNEATGGK can be used as safe ACE inhibitors in the concentration range of 0.001 to 0.25 mg/mL without significant cytotoxicity to the endothelial cells [35].

#### 2.6.2. Effect of Se-Enriched Oyster-Derived ACE Inhibitory Peptides on NO Secretion of Endothelial Cells

**Figure 5:**
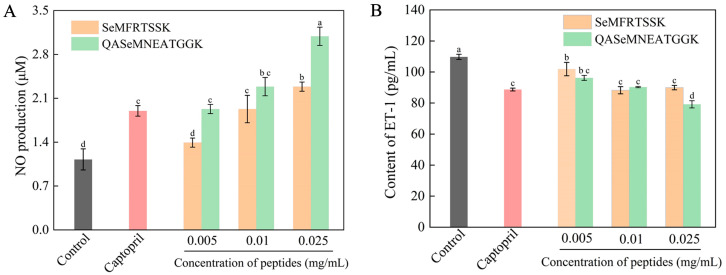
Effect of Se-enriched oyster-derived ACE inhibitory peptides on the NO production (**A**) and the ET-1 secretion (**B**) of EA.hy 926 cells. Different letters in the same test indicate significant differences (*p* < 0.05).

In this work, endothelial cell EA.hy926, a typical cell evaluation model for antihypertensive activity, was employed [36]. The production of NO from cells was used as an index of antihypertensive activity since NO can relax vascular smooth muscle, balance human blood pressure, and maintain constant vascular tone [36]. The EA.hy926 cells were treated with Se-enriched oyster-derived ACE inhibitory peptides at different concentrations, and captopril was used for the positive control cells. The NO production of all cell groups was measured by the Griess assay and is shown in Figure 5A. The cell groups treated with SeMFRTSSK and QASeMNEATGGK showed a higher content of NO compared to the blank control group, and the NO content increased as the peptide concentrations increased. At a concentration of 0.025 mg/mL (approximately 27.7 μM for SeMFRTSSK and 23.7 μM for QASeMNEATGGK), NO production in cells treated with SeMFRTSSK and QASeMNEATGGK increased by 202.65% and 273.45%, respectively, compared to the blank control group. This NO production was also significantly higher than that of the captopril positive control group (2.29 ± 0.07 μM for SeMFRTSSK and 3.09 ± 0.15 μM for QASeMNEATGGK vs. 1.89 ± 0.08 μM for captopril). Clearly, SeMFRTSSK and QASeMNEATGGK could promote the NO production of EA.hy926 cells and exert antihypertensive function with a dose effect.

#### 2.6.3. Effect of Se-Enriched Oyster-Derived ACE Inhibitory Peptides on ET-1 Secretion of Endothelial Cell

In addition to the renin angiotensin system, the endothelin system has been increasingly recognized as playing a role in the regulation of blood pressure, in which, ET-1 exerts a strong function in vasoconstriction and elevation of blood pressure [37]. Generally, a lower level of ET-1 secretion implies a higher performance in ACE inhibition [38]. In this work, EA.hy926 cells were treated with Se-enriched oyster-derived ACE inhibitory peptides at different concentrations, and the antihypertensive activity of the targeted peptides was evaluated by the level of ET-1 secretion. As shown in Figure 5B, after 24 h of treatment, the treated cell groups revealed a lower release of ET-1 compared to the blank control group. Meanwhile, the ET-1 release from the treated cell groups exhibited a significant dose dependence. In the high-dose group (0.025 mg/mL, ~27.7 μM), the SeMFRTSSK-treated system featured an ET-1 content of 88.94 ± 1.41 pg/mL, which was almost identical to the ET-1 content of the captopril positive control group (88.67 ± 0.91 pg/mL) (*p* < 0.05). The ET-1 content for the QASeMNEATGGK-treated group was 79.15 ± 2.32 pg/mL, which was significantly lower than that of the positive control group (109.72 ± 1.66 pg/mL). These results substantiate that the targeted ACE inhibitory peptides can exert a hypotensive effect by inhibiting the expression of ET-1 in EA.hy926 cells.

Taken together, SeMFRTSSK and QASeMNEATGGK could promote the NO production and inhibit the ET-1 secretion in endothelial cells while delivering negligible cytotoxicity at concentrations less than 0.25 mg/mL. These results indicated the potential of oyster-derived Se-enriched ACE inhibitory peptides in the hypertension management by increasing NO release and restraining ET-1 secretion.

## 3. Materials and Methods

### 3.1. Materials and Chemicals

The oyster utilized in this work was produced in the Maowei Sea, China. This oyster was thoroughly washed with water, freeze-dried, and ground into particles with a size smaller than 0.15 mm. The Se content of the oyster powder was tested to be 1.30 ± 0.07 mg/kg, classifying it as a Se-enriched product. Alcalase (2 × 10^5^ U/g), neutrase (2 × 10^5^ U/g), papain (2 × 10^5^ U/g), and trypsin (2500 Usp U/mg) were purchased from Nanning Pangbo Biological Engineering Co., Ltd. located in Nanning, Guangxi, China. ACE (isolated from rabbit lung) and N-Hippuryl-His-Leu hydrate (HHL) were provided by Sigma-Aldrich (St. Louis, MO, USA). 3-(4,5-dimethylthiazol-2-yl)-2,5 diphenyl tetrazolium bromide (MTT) was obtained from Shanghai Maclean Biochemical Technology Co., Ltd. (Shanghai, China). Dulbecco’s modified Eagle’s medium (DMEM), fetal bovine serum (FBS), and penicillin/streptomycin were purchased from Gibco BRL Life Technology (Gaithersburg, MD, USA). Nitric oxide assay kit was purchased from Beyotime Biotechnology Co., Ltd. (Nanjing, China). The uman Endothelin 1 (ET-1) ELISA kit was offered by CUSABIO (Wuhan, China).

### 3.2. Preparation of Se-Enriched Oyster Hydrolysate

Under the optimal conditions recommended by the production enterprise, a single enzyme was used for enzymatically hydrolyzing the Se-enriched oyster powder. Specifically, for alcalase (55 °C, pH 8.5), neutrase (45 °C, pH 7.0), papain (55 °C, pH 6.5), and trypsin (37 °C, pH 8.0). Generally, the Se-enriched oyster powder was dissolved in deionized water at a solid-liquid ratio of 5 g/100 mL and the pH of the mixture was adjusted to the recommended value by using a HCl or NaOH solution (0.1 mol/L). Subsequently, the protease was added to the mixture with an enzyme/substrate ratio of 0.3%. After incubating for 3.0 h at the recommended temperature, the hydrolysate solution was heated at 95 °C for 10 min to terminate the hydrolysis reaction. The resulting samples were cooled down to room temperature and centrifuged for 20 min at 4000 rpm. The supernatants, namely the Se-enriched oyster hydrolysates, were collected, freeze-dried, and stored at −20 °C for subsequent analysis.

### 3.3. Assay for ACE Inhibitory Activity and IC_50_

ACE inhibitory activity was determined in accordance with the method reported by Tu et al. with slight modifications [39]. Briefly, the ACE inhibitory peptide (ACEI) solution was prepared by dissolving the Se-enriched oyster-derived peptides in 0.1 mol/L sodium borate buffer (pH 8.3, 0.3 mol/L NaCl). The ACE solution (10 μL, 0.1 U/mL) and the ACEI solution (10 μL) were mixed and heated for 5 min in a 37 °C water bath. The HHL solution (30 μL, 5.0 mmol/L) was added to the mixture and held for another 1.0 h at 37 °C. Subsequently, 80 μL of HCl solution (1.0 mol/L) was added under oscillation to terminate the reaction. The resultant solution was cooled down to room temperature and used for the analysis of hippuric acid (HA). In the control group, the ACEI solution was replaced by 0.1 mol/L sodium borate buffer (10 μL, pH 8.3, 0.3 mol/L NaCl). For the blank group, prior to the addition of ACE, 80 μL of HCl solution (1.0 mol/L) was used to inactivate the Se-enriched oyster-derived peptide.

The resulting solution was passed through a 0.45 μm membrane and then analyzed using a high-performance liquid chromatography (HPLC) equipped with a Welch Ultimate LC-C18 (HS) column (250 mm × 4.6 mm, 5 µm particle size). The elution program was set as follows: 25% mobile phase B (ACN) and 75% mobile phase A (0.1% trifluoroacetic acid in H_2_O, *v*/*v*), with a flow rate of 1.0 mL/min, a UV detection wavelength of 228 nm, a column temperature of 30 °C, and an injection volume of 20 μL. The ACE inhibitory activity (%) was calculated according to the following equation [39].

$$\mathrm{ACE}\;\mathrm{inhibitory}\;\mathrm{activity}\;\left(\%\right)=100\%\times \left(A-B-A_{0}\right)/\left(A-A_{0}\right)$$

where A, B and A_0_ represent the peak areas of HA in the control group, the sample and the blank group, respectively.

SPSS 26.0 software was used to analyze the ACE inhibitory activity of Se-enriched oyster-derived peptides at different concentrations, and the half maximal inhibitory concentration (IC_50_) of the peptides was determined via the regression equation.

### 3.4. Purification of Se-Enriched Oyster-Derived ACE Inhibitory Peptides

#### 3.4.1. Ultrafiltration

Based on molecular weight, the Se-enriched oyster-derived hydrolysates were separated by using an ultrafiltration tube (Amicon Ultra-15, 10 kDa; Merck Millipore, Burlington, MA, USA) under a 30-min centrifugation at 4000 rpm. The sections with molecular weight (MW) less than 10 kDa were selected and stored in a −20 °C refrigerator.

#### 3.4.2. Preparative Reversed-Phase High-Performance Liquid Chromatography (RP-HPLC)

In the preparative RP-HPLC system (LC-8, Shimadzu, Japan), the ultrafiltration-derived components (MW < 10 kDa) were separated by using a PRC-ODS (K) column (30 mm × 250 mm, 15 µm, Shimadzu, Kyoto, Japan). The chromatographic conditions were as follows: mobile phase A was 0.1% trifluoroacetic acid in water and mobile phase B was 0.1% trifluoroacetic acid in methanol. The elution procedure time was set as follows: 0–45 min, 5–10% mobile phase B; 45–65 min, 10–20% mobile phase B; 65–75 min, 20–50% mobile phase B; 75–85 min, 95–95% mobile phase B; 85–90 min, 95–5% mobile phase B. The injection volume was 5 mL and the eluent at a flow rate of 10 mL/min was identified at 214 nm and 280 nm. The eluents from the same elution peak were collected, concentrated, and freeze-dried. The resultant sections were then employed for the analysis of ACE inhibitory activity.

A preparative RP-HPLC system (Prep 150) equipped with a SunFire Prep C18 OBD™ column (19 mm × 250 mm, 5 µm, Waters, Milford, MA, USA) was utilized to further isolate the section with the highest ACE inhibitory activity. The chromatographic conditions were as follows: mobile phase A was 0.1% trifluoroacetic acid solution; mobile phase B was 0.1% trifluoroacetic acid in methanol. Gradient elution was set as follows: 0–15 min, 6–8% mobile phase B; 15–20 min, 8–40% mobile phase B; 20–40 min, 40–50% mobile phase B; 40–50 min, 95–95% mobile phase B; 50–55 min, 95–6% mobile phase B. The flow rate was 10 mL/min and the detection wavelength was 214 nm. The active fractions were collected, concentrated by rotary evaporation and freeze-dried. Then, the ACE inhibitory activity for each section was determined, and the fraction with the highest ACE inhibitory activity was further identified by mass spectrometry.

### 3.5. Analysis of the Se Content

The Se contents of the samples were determined in accordance with the Chinese national standard method GB 5009.93-2017 [40], using an atomic fluorescence spectrophotometer (AFS-9530, Beijing Haiguang Instruments Co., Ltd., Beijing, China).

### 3.6. Characterization of the Se-Enriched Oyster-Derived ACE Inhibitory Peptide

The online LC/MS analysis was employed to characterize the peptide structure according to the previously established method [41]. Briefly, the fraction with the highest ACE inhibition capacity (M4-2) was first dissolved in deionized water containing 0.1% formic acid and then desalinated using a C18 ZipTip (Acclaim PepMap 100, 75 μm × 25 cm; Thermo Fisher Scientific, Waltham, MA, USA). The resultant sample was subjected to an online nanoliter liquid phase system (Easy nLC 1200, Thermo Fisher Scientific, Waltham, MA, USA) coupled with a Q Exactive™ Plus mass spectrometer (ThermoFisher). The spray voltage and the interface heater temperature were set at 1.9 kV and 275 °C, respectively. The elution gradient of mobile phase B (80% acetonitrile, 0.1% formic acid) increased from 5% to 38% within 60 min. The obtained MS/MS data were analyzed on PEAKS Studio 8.5 (Bioinformatics Solutions Inc., Waterloo, ON, Canada). The peptide sequences with ALC ≥ 60% were selected for further analysis.

### 3.7. Molecular Docking Simulation

The RCSB Protein Data Bank (www.rcsb.org (accessed on 20 October 2022)) was consulted to obtain the crystal structure of the receptor molecule, 1O8A protein. The PyMol software (version 2.5.0; Schrödinger, LLC, New York, NY, USA) was used to remove the water molecules and the original ligands in the receptor molecule. A positive charge of 0.95 e was added to the Zn^2+^ in ACE. The resultant receptor molecule was saved as a PDB file. This receptor molecule was then hydrogen-treated in the Autodock Tools 1.5.6 software and saved as a PDBQT file for further use. The Marvin Sketch software (version 22.17.0; ChemAxon Ltd., Budapest, Hungary) was employed to draw and optimize the 2D structures of the ligand molecules to achieve the minimum energy [42], and then the corresponding 3D structures were output in a mol2 format file. This file was opened in the Autodock Tools software, and the ligand molecules underwent hydrogen treatment, Gasteiger calculation, and rotation key setting. The resultant structures were saved in a PDBQT file. Herein, the dimensions of Grid Box (x, y, z) were set as (100.125, 100.125, 100.125) with a center of (43.821, 38.24, 46.712), and the exhaustiveness value was 20 [43]. The docking program for the receptor and ligand was conducted on Autodock Vina 1.1.2 using the built-in scoring function [44]. Finally, the molecular docking results were visually analyzed on the Discovery Studio 4.5 software, and the conformation with the lowest binding energy was selected as the optimal binding site.

### 3.8. Synthesis of ACE Inhibitory Peptides

Two novel Se-enriched oyster-derived ACE inhibitory peptides were artificially synthesized by Synpeptide Co., Ltd. (Nanjing, China). The purity of these peptides was verified to be >98% on HPLC.

### 3.9. Cell Culture and Cell Viability Assay

EA.hy926 cells were cultured according to the previously established method by Zheng et al. with some modifications [45]. Typically, EA.hy926 cells were first inoculated onto the DMEM supplemented with 20% FBS and 1% penicillin/streptomycin in an incubator (5% CO_2_, 37 °C). The resultant EA.hy926 cells were seeded into a 96-well plate (1 × 10^5^ cells/100 μL/well) for an adherent culture of 24 h; after which the culture medium was discarded. In the peptide treatment group, cells were incubated with 100 µL of Se-enriched oyster-derived ACE inhibitory peptide at a series of concentrations (0.001, 0.005, 0.01, 0.025, 0.05, 0.1, 0.25, and 0.5 mg/mL). The blank control group was incubated with the medium, while the positive control group was incubated with captopril. After a culture of 24 h, the cell viability was determined by the MTT assay as described by Mosmann [46]. Briefly, the resultant cells were washed with PBS and then incubated for another 4 h under 100 µL of MTT solution (0.5 mg/mL). Thereafter, the MTT solution was replaced by 100 µL of DMSO and oscillated for 10 min to fully dissolve the blue-violet crystals. The absorbance at 490 nm was measured to evaluate the cell viability using a microplate reader (EnSpire 2300, PerkinElmer, Waltham, MA, USA). 

### 3.10. Determination of NO and ET-1 Release from Cells

EA.hy926 cells were seeded in 96-well plates at a density of 1 × 10^5^ cells/well. After a 24-h cultivation at 37 °C, the culture medium was removed. In the blank control group, 100 μL of culture medium was added. In the peptide treatment groups, 100 μL of Se-enriched oyster-derived ACE inhibitory peptide solutions with different concentrations, such as 0.005, 0.01, and 0.025 mg/mL, were added. For the positive control group, 100 μL of 0.5 mg/mL Captopril solution was used. Each experimental condition was performed with 2 independent biological replicates (*n* = 2), and each detection was carried out with 3 technical replicates. All cell groups were incubated for another 24 h. Subsequently, the cell culture medium was collected from each well and subjected to the NO assay kit and ET-1 ELISA kit, respectively, for determining the contents of NO and ET-1. Captopril at 0.5 mg/mL (~2.3 mM) was used as a high-concentration positive control to validate the assay system and establish a benchmark for maximum inhibition.

### 3.11. Statistical Analysis

All assays were performed in triplicate, and the results were expressed as mean ± standard deviation. One-way analysis of variance (ANOVA) and significance analysis were conducted using SPSS 26.0 and the Duncan’s test, respectively. A *p* value less than 0.05 was regarded as a significant difference with statistical significance.

## 4. Conclusions

In summary, a fraction (M4-2) with the highest ACE inhibitory activity was isolated from Se-enriched oyster hydrolysates via ultrafiltration and a two-step RP-HPLC purification process. The M4-2 fraction featured a significantly higher Se content than both the parent hydrolysate and the original oyster powder. This fraction was characterized by LC-MS/MS and revealed 91 Se-enriched peptide sequences. Based on the molecular docking, two novel selenium-containing peptides, SeMFRTSSK (903.3717 Da) and QASeMNEATGGK (1053.3994 Da), which showed the strongest affinity towards ACE, were obtained. These two peptides could form stable complexes with ACE via distinct binding modes and sites. SeMFRTSSK interacted with amino acid residues Ala354, Glu384, Tyr523, Gln281, His353, Lys511, and His513 in the active pocket of ACE through strong hydrogen bonds to exert ACE inhibitory activity. However, the binding of QASeMNEATGGK to Glu411 and Zn701 sites in ACE might play a key role in ACE inhibition. Moreover, endothelial cell assays supported the very low cytotoxicity and the effective cellular antihypertensive activity of SeMFRTSSK and QASeMNEATGGK. These results indicated the potential of SeMFRTSSK and QASeMNEATGGK as functional components for antihypertension. This study suggests a promising strategy for the high-value utilization of Se-enriched oysters in hypertension management.

## Figures and Tables

**Table 1:** Se content and IC_50_ of ACE inhibitory activity for hydrolysate and M4-2.

Sample	Total Se Content (mg/kg)	IC_50_ (mg/mL)
Hydrolysate	2.44 ± 0.71	2.801
M4-2	37.00 ± 0.56	0.774

**Table 2:** Selected ACE inhibitory peptides from the fraction M4-2 identified by LC-MS/MS analysis with the ALC ≥ 60%.

Number	Peptide	ALC (%)	Length	Mass (Da)	Affinity (kcal/mol)
1	SeMFRTSSK	60	7	903.3717	−9.8
2	QASeMNEATGGK	78	10	1053.3994	−9.0
3	KFGSeCGAK	73	7	757.3026	−8.9
4	KFSeCAAK	64	6	714.2968	−8.9
5	TTSeCTAPR	61	7	796.2982	−8.9
6	YSeMVDASK	70	7	860.3183	−8.8
7	SeMFAVQGWNK	70	9	1127.4668	−8.8
8	KSSeCASVPR	64	8	894.3826	−8.8
9	HSeCSeCAAK	75	6	727.1459	−8.7
10	AHGGASeCR	65	7	718.2414	−8.7
11	TSSeCAAR	65	6	655.2192	−8.7
12	QYSeMEENK	62	7	988.3405	−8.7
13	FSeMDLR	60	5	728.2760	−8.7
14	DSSeCAR	79	5	598.1614	−8.6
15	DHFSeCELGGK	63	9	1052.3831	−8.6
16	QLDSSeMR	71	6	796.2982	−8.5
17	QFSeMVK	64	5	699.2859	−8.5
18	FLSeMEK	88	5	714.2855	−8.4
19	TSeMMVWSK	66	7	929.3584	−8.4
20	ARFSeCGDK	65	7	843.3141	−8.4
21	DHSeMEQR	64	6	862.2836	−8.4
22	SGGASeCR	63	6	597.1774	−8.4
23	FLSeMEAK	85	6	785.3226	−8.3
24	YNFRSeCGK	76	7	934.3564	−8.3
25	HPSeMLKK	66	6	800.3812	−8.3
26	LSGSeCWHK	64	7	877.3350	−8.3
27	LWSeMK	62	4	624.2538	−8.3
28	TSAAGSeCEK	90	8	813.2772	−8.2
29	HSeCPLSEK	80	7	860.3295	−8.2
30	SVAGSeMK	73	6	639.2495	−8.2
31	QSeMANEATGGK	73	10	1053.3994	−8.2
32	FSeMLAK	69	5	656.2800	−8.2
33	QSeMASTDANK	69	9	1012.3729	−8.2
34	TRSSeMR	68	5	697.2774	−8.2
35	TSSeCTVKPK	68	8	910.4026	−8.2
36	TSeMMKESR	64	7	929.3544	−8.2
37	LSeMLR	78	4	579.2647	−8.1
38	QTSeMWTNSK	73	8	1042.3987	−8.1
39	FSeCSeCAK	71	5	666.1183	−8.1
40	QGASeCR	68	5	581.1824	−8.1
41	ASeCGNELR	67	7	809.2935	−8.1
42	KSeCYTAK	66	6	760.3022	−8.1
43	KFSeCGGK	63	6	686.2654	−8.1
44	SeMPHDDGHVEK	76	10	1211.4475	−8
45	TLSeCMSPEHSSSSK	83	13	1440.5459	−8
46	ESeMGGDK	81	6	683.2029	−8
47	LFSeMALK	71	6	769.3641	−8
48	RSeCVDAK	68	6	738.2927	−8
49	ADSeMPK	66	5	608.2073	−8
50	EDASeCAK	62	6	683.2029	−8
51	VDKSeCAK	82	6	710.2866	−7.9
52	FLSeMK	77	4	585.2429	−7.9
53	YSeMLSTDLR	72	8	1045.4348	−7.9
54	VPPSeCK	71	5	590.2331	−7.9
55	KGSeCSeCPTLR	70	8	972.3199	−7.9
56	DNSeMALK	66	6	738.2815	−7.9
57	AFSeCLR	61	5	656.2549	−7.9
58	TSSSeMLTK	71	7	814.3339	−7.8
59	STTTSeMK	69	6	715.2656	−7.8
60	QSLSeCLK	67	6	738.3179	−7.8
61	ELSeMPK	60	5	664.2699	−7.8
62	FSeCQLAK	87	6	756.3073	−7.7
63	TTSeMSENDAPK	77	10	1140.4202	−7.7
64	QTSeMWENK	69	7	983.3616	−7.7
65	TVSeCPPK	69	6	691.2808	−7.7
66	RSeCADGK	65	6	696.2458	−7.7
67	KPNTTSeCK	62	7	838.3452	−7.7
68	KSSeCAR	68	5	611.2294	−7.6
69	QSeCNELR	66	6	809.2935	−7.6
70	KFNSeCNK	60	6	800.3084	−7.6
71	FSeMALK	78	5	656.2800	−7.4
72	GSeCSeCSQK	74	6	720.1249	−7.4
73	WTSeCAK	73	5	655.2233	−7.4
74	KSeMVDAK	70	6	738.3179	−7.4
75	TSKSeCAK	65	6	684.2709	−7.4
76	YSeCSeCAK	64	5	682.1132	−7.4
77	EGTAKSeCR	60	7	811.3091	−7.4
78	GSSLSeCK	67	6	641.2288	−7.3
79	VMSeMLK	63	5	668.2834	−7.3
80	LTSeCLR	77	5	652.2811	−7.2
81	TSeMLK	76	4	539.2222	−7.2
82	NGSeCAK	75	5	539.1606	−7.2
83	KFASeCGK	75	6	700.2811	−7.2
84	ATSeMLR	69	5	638.2654	−7.2
85	SSLTSeMK	84	6	713.2863	−7.1
86	QTSKSeMK	62	6	769.3237	−7
87	NSSVSeMK	63	6	712.2659	−7
88	RGNSeCAK	76	6	695.2618	−6.9
89	MGTLLSSeCK	67	8	899.3690	−6.9
90	RTSeCAK	69	5	625.2451	−6.8
91	SSLSeMK	68	5	612.2386	−6.7

**Table 3:** Interactions and active sites of Se-enriched oysters-derived ACE inhibitory peptides and ACE.

Peptide	Hydrogen Bond	Hydrophobic Interaction	Electrostatic Interaction	Metal- Acceptor
SeMFRTSSK	Gln281 Thr282 Asn277 Cys352 His353 Ala354 Cys370 Glu384 Asp377 Lys511 Tyr523 His513	Thr166 Val379 Phe527	Glu162 Glu376 Asp377 His410 Asp453	
QASeMNEATGGK	Trp59 Tyr62 Asn66 Asn70 Leu139 Ala356 His410 Glu411 Pro515	Val518 Pro519		Zn701

## Data Availability

The original contributions presented in this study are included in the article. Further inquiries can be directed to the corresponding authors.

## Supplementary Materials

The following supporting information can be downloaded at: https://www.mdpi.com/article/10.3390/molecules30244818/s1, Table S1: Complete list of the 91 selenium-containing peptides identified from the M4-2 fraction with ALC ≥ 60%, grouped by the physicochemical property of their N-terminal residue and sorted by binding affinity within each group; Figure S1: HPLC chromatographic profile of the purified fraction containing peptide SeMFRTSSK; Figure S2: Mass spectrometric characterization of peptide SeMFRTSSK; Figure S3: HPLC chromatographic profile of the purified fraction containing peptide QASeMNEATGGK; Figure S4: Mass spectrometric characterization of peptide SeMFRTSSK.
