# Supplementary material for: Identification, Molecular Docking Mechanism and Cellular Activity of Selenium-Enriched ACE Inhibitory Peptides from Oysters
Source: Molecules. 2025 Dec 18;30(24):4818. doi: 10.3390/molecules30244818 (PMC12735907; doi:10.3390/molecules30244818)

Supporting Information for

Identification, Molecular Docking Mechanism and Cellular Activity of Selenium-Enriched ACE Inhibitory Peptides from Oysters

Zhuangzhuang Yue [1,†], Zhen Xia [2,†], Fei Xu [1], Bingbing Chen [2], Shufei Jiao [1], Xingtang Liang [1,*], Yanzhen Yin [1], Jianyin Miao [2,*]

1 Guangxi Key Laboratory of Green Chemical Materials and Safety Technology, School of Petroleum and Chemical Engineering, Beibu Gulf University, Qinzhou 535011, China

2 Guangdong Provincial Key Laboratory of Nutraceuticals and Functional Foods, College of Food Science, South China Agricultural University, Guangzhou 510642, China

* Correspondence: liangxingtang@bbgu.edu.cn (X.L.); miaojy8181@scau.edu.cn (J.M.)

† These authors contributed equally to this work

Table S1.

Complete list of the 91 selenium-containing peptides identified from the M4-2 fraction with ALC ≥ 60%, grouped by the physicochemical property of their N-terminal residue and sorted by binding affinity within each group.

I. Peptides with a hydrophobic N-terminal residue (A, F, I, L, M, V, W, Y) [Total: 36 peptides]

No.	Peptide Sequence	ALC (%)	Length	Mass (Da)	Affinity (kcal/mol)
1	SeMFRTSSK	60	7	903.3717	-9.8
7	SeMFAVQGWNK	70	9	1127.4668	-8.8
6	YSeMVDASK	70	7	860.3183	-8.8
13	FSeMDLR	60	5	728.2760	-8.7
17	QFSeMVK*	64	5	699.2859	-8.5
18	FLSeMEK	88	5	714.2855	-8.4
23	FLSeMEAK	85	6	785.3226	-8.3
32	FSeMLAK	69	5	656.2800	-8.2
37	LSeMLR	78	4	579.2647	-8.1
44	SeMPHDDGHVEK	76	10	1211.4475	-8.0
47	LFSeMALK	71	6	769.3641	-8.0
52	FLSeMK	77	4	585.2429	-7.9
53	YSeMLSTDLR	72	8	1045.4348	-7.9
57	AFSeCLR	61	5	656.2549	-7.9
62	FSeCQLAK	87	6	756.3073	-7.7
71	FSeMALK	78	5	656.2800	-7.4
79	VMSeMLK	63	5	668.2834	-7.3
81	TSeMLK*	76	4	539.2222	-7.2

84	ATSeMLR	69	5	638.2654	-7.2
15	DHFSeCELGGK*	63	9	1052.3831	-8.6
56	DNSeMALK*	66	6	738.2815	-7.9
54	VPPSeCK*	71	5	590.2331	-7.9
80	LTSeCLR*	77	5	652.2811	-7.2
83	KFASeCGK*	75	6	700.2811	-7.2
24	YNFRSeCGK*	76	7	934.3564	-8.3
27	LWSeMK*	62	4	624.2538	-8.3
39	FSeCSeCAK*	71	5	666.1183	-8.1
76	YSeCSeCAK*	64	5	682.1132	-7.4
72	GSeCSeCSQK*	74	6	720.1249	-7.4
73	WTSeCAK*	73	5	655.2233	-7.4
75	TSKSeCAK*	65	6	684.2709	-7.4
77	EGTAKSeCR*	60	7	811.3091	-7.4
86	QTSKSeMK*	62	6	769.3237	-7.0
60	QSLSeCLK*	67	6	738.3179	-7.8
61	ELSeMPK*	60	5	664.2699	-7.8
65	TVSeCPPK*	69	6	691.2808	-7.7

II. Peptides with a polar neutral N-terminal residue (C, N, Q, S, T) [Total: 33 peptides]

No.	Peptide Sequence	ALC (%)	Length	Mass (Da)	Affinity (kcal/mol)
2	QASeMNEATGGK	78	10	1053.3994	-9.0
5	TTSeCTAPR	61	7	796.2982	-8.9
10	AHGGASeCR	65	7	718.2414	-8.7

11	TSSeCAAR	65	6	655.2192	-8.7
12	QYSeMEENK	62	7	988.3405	-8.7
16	QLDSSeMR	71	6	796.2982	-8.5
19	TSeMMVWSK	66	7	929.3584	-8.4
22	SGGASeCR	63	6	597.1774	-8.4
28	TSAAGSeCEK	90	8	813.2772	-8.2
30	SVAGSeMK	73	6	639.2495	-8.2
31	QSeMANEATGGK	73	10	1053.3994	-8.2
33	QSeMASTDANK	69	9	1012.3729	-8.2
34	TRSSeMR	68	5	697.2774	-8.2
35	TSSeCTVKPK	68	8	910.4026	-8.2
36	TSeMMKESR	64	7	929.3544	-8.2
38	QTSeMWTNSK	73	8	1042.3987	-8.1
40	QGASeCR	68	5	581.1824	-8.1
41	ASeCGNELR	67	7	809.2935	-8.1
45	TLSeCMSPEHSSSSK	83	13	1440.5459	-8.0
46	ESeMGGDK*	81	6	683.2029	-8.0
49	ADSeMPK	66	5	608.2073	-8.0
50	EDASeCAK*	62	6	683.2029	-8.0
58	TSSSeMLTK	71	7	814.3339	-7.8
59	STTTSeMK	69	6	715.2656	-7.8
63	TTSeMSENDAPK	77	10	1140.4202	-7.7
64	QTSeMWENK	69	7	983.3616	-7.7
78	GSSLSeCK	67	6	641.2288	-7.3
82	NGSeCAK	75	5	539.1606	-7.2

85	SSLTSeMK	84	6	713.2863	-7.1
87	NSSVSeMK	63	6	712.2659	-7.0
89	MGTLLSSeCK	67	8	899.3690	-6.9
90	RTSeCAK*	69	5	625.2451	-6.8
91	SSLSeMK	68	5	612.2386	-6.7

III. Peptides with a positively charged N-terminal residue (H, K, R) [Total: 22 peptides]

No.	Peptide Sequence	ALC (%)	Length	Mass (Da)	Affinity (kcal/mol)
3	KFGSeCGAK	73	7	757.3026	-8.9
4	KFSeCAAK	64	6	714.2968	-8.9
8	KSSeCASVPR	64	8	894.3826	-8.8
9	HSeCSeCAAK	75	6	727.1459	-8.7
14	DSSeCAR*	79	5	598.1614	-8.6
20	ARFSeCGDK*	65	7	843.3141	-8.4
21	DHSeMEQR*	64	6	862.2836	-8.4
25	HPSeMLKK	66	6	800.3812	-8.3
26	LSGSeCWHK*	64	7	877.3350	-8.3
29	HSeCPLSEK	80	7	860.3295	-8.2
42	KSeCYTAK	66	6	760.3022	-8.1
43	KFSeCGGK	63	6	686.2654	-8.1
48	RSeCVDAK	68	6	738.2927	-8.0
51	VDKSeCAK*	82	6	710.2866	-7.9
55	KGSeCSeCPTLR	70	8	972.3199	-7.9
66	RSeCADGK	65	6	696.2458	-7.7

67	KPNTTSeCK	62	7	838.3452	-7.7
68	KSSeCAR	68	5	611.2294	-7.6
69	QSeCNELR*	66	6	809.2935	-7.6
70	KFNSeCNK	60	6	800.3084	-7.6
74	KSeMVDAK	70	6	738.3179	-7.4
88	RGNSeCAK*	76	6	695.2618	-6.9

Notes:
SeM denotes selenomethionine.
Grouping principle: Peptides are grouped based on the common side-chain property (at neutral pH) of their N-terminal amino acid residue. Residues are categorized as: Hydrophobic (A, F, I, L, M, V, W, Y), Polar Neutral (C, N, Q, S, T), or Positively Charged (H, K, R).
Asterisk (*): For peptides marked with an asterisk, the N-terminal residue (e.g., D, E, G, etc.) is classified based on its interaction context in the present docking study or its modified state, rather than its standard net charge at pH 7, to facilitate structural trend analysis within the grouped dataset.
Sorting: Within each group, peptides are sorted in descending order of binding affinity (most negative to least negative kcal/mol value) to ACE.

Figure S1:

HPLC chromatographic profile of the purified fraction containing peptide SeMFRTSSK.

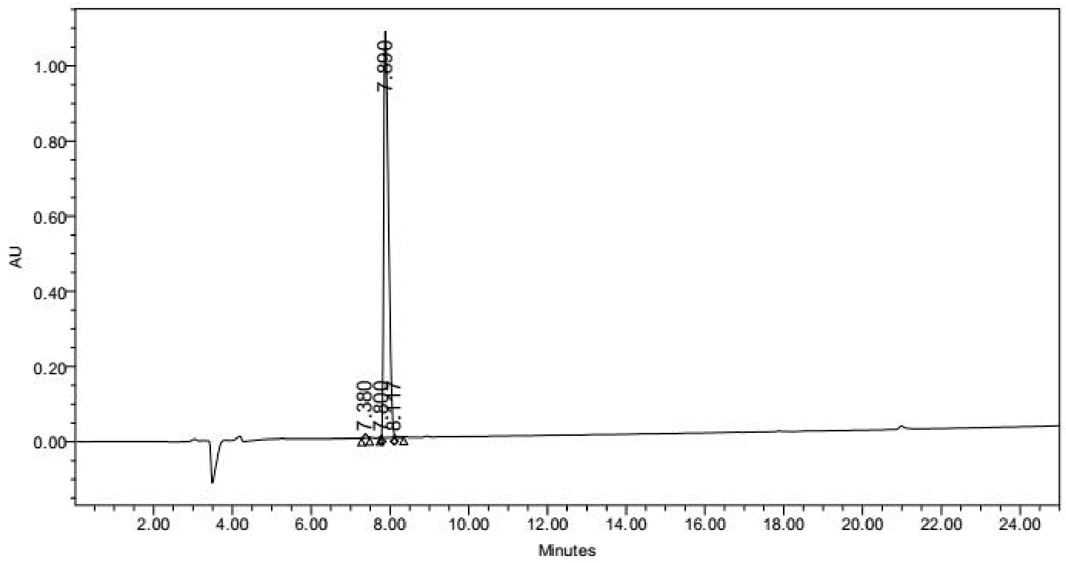

	RT	Area	% Area	Height
1	7.380	50452	0.59	9627
2	7.800	3555	0.04	6547
3	7.890	8433276	99.14	1089989
4	8.117	19572	0.23	6520

Figure S2:

Mass spectrometric characterization of peptide SeMFRTSSK.

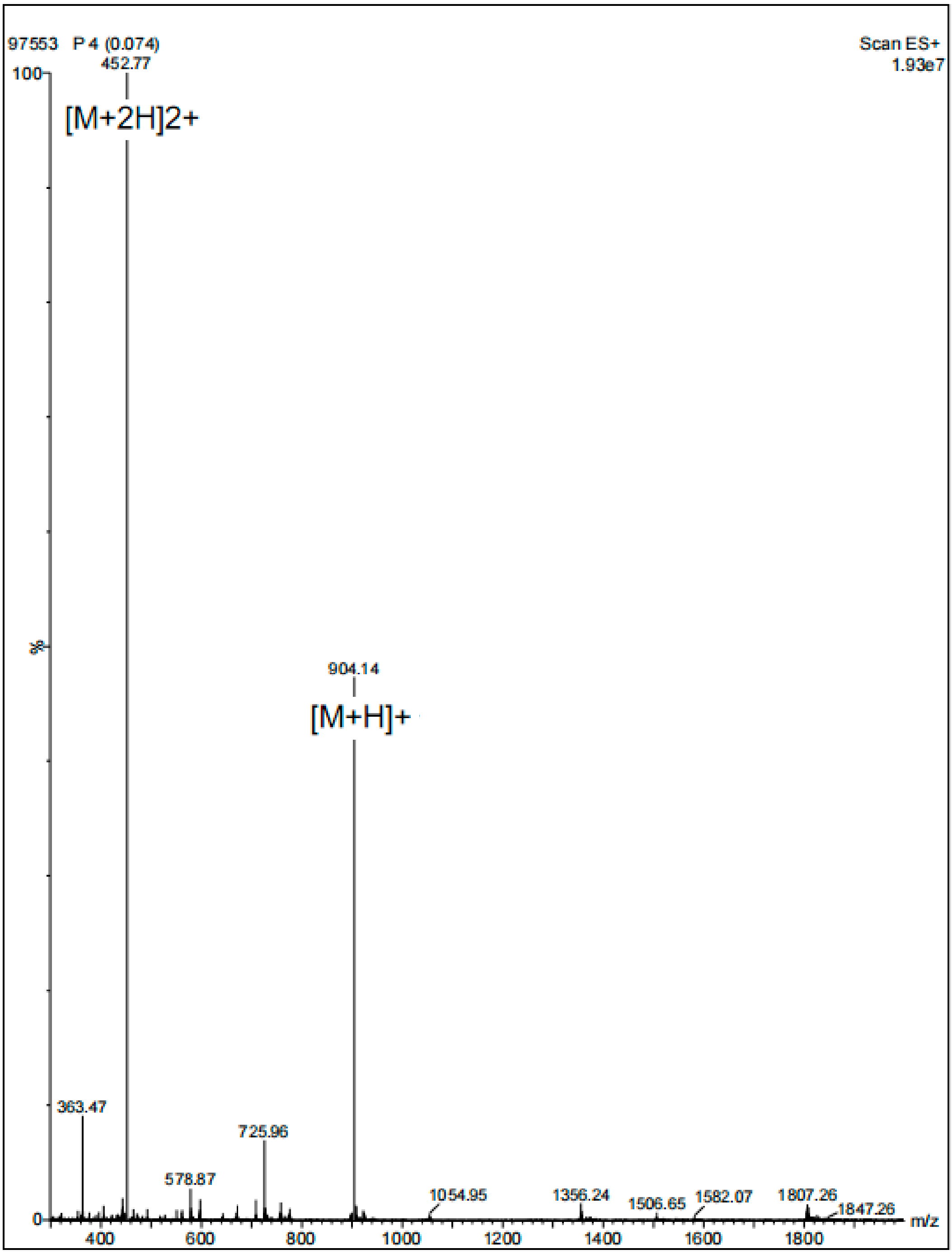

Figure S3:

HPLC chromatographic profile of the purified fraction containing peptide QASeMNEATGGK.

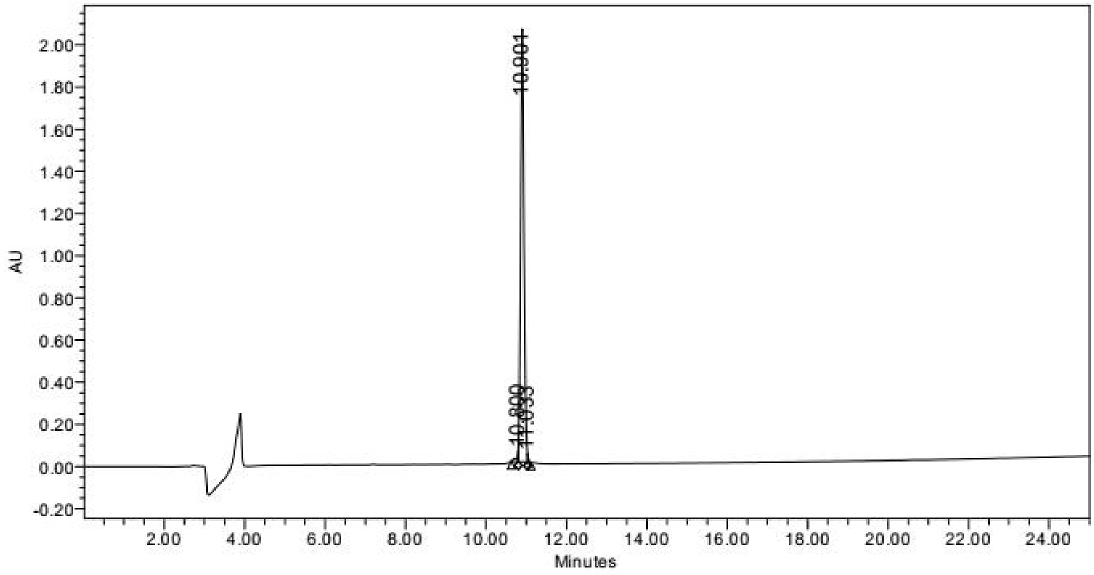

	RT	Area	% Area	Height
1	10.800	125686	1.00	48636
2	10.901	12366536	98.58	2042071
3	11.033	52698	0.42	38739

Figure S4:

Mass spectrometric characterization of peptide SeMFRTSSK.

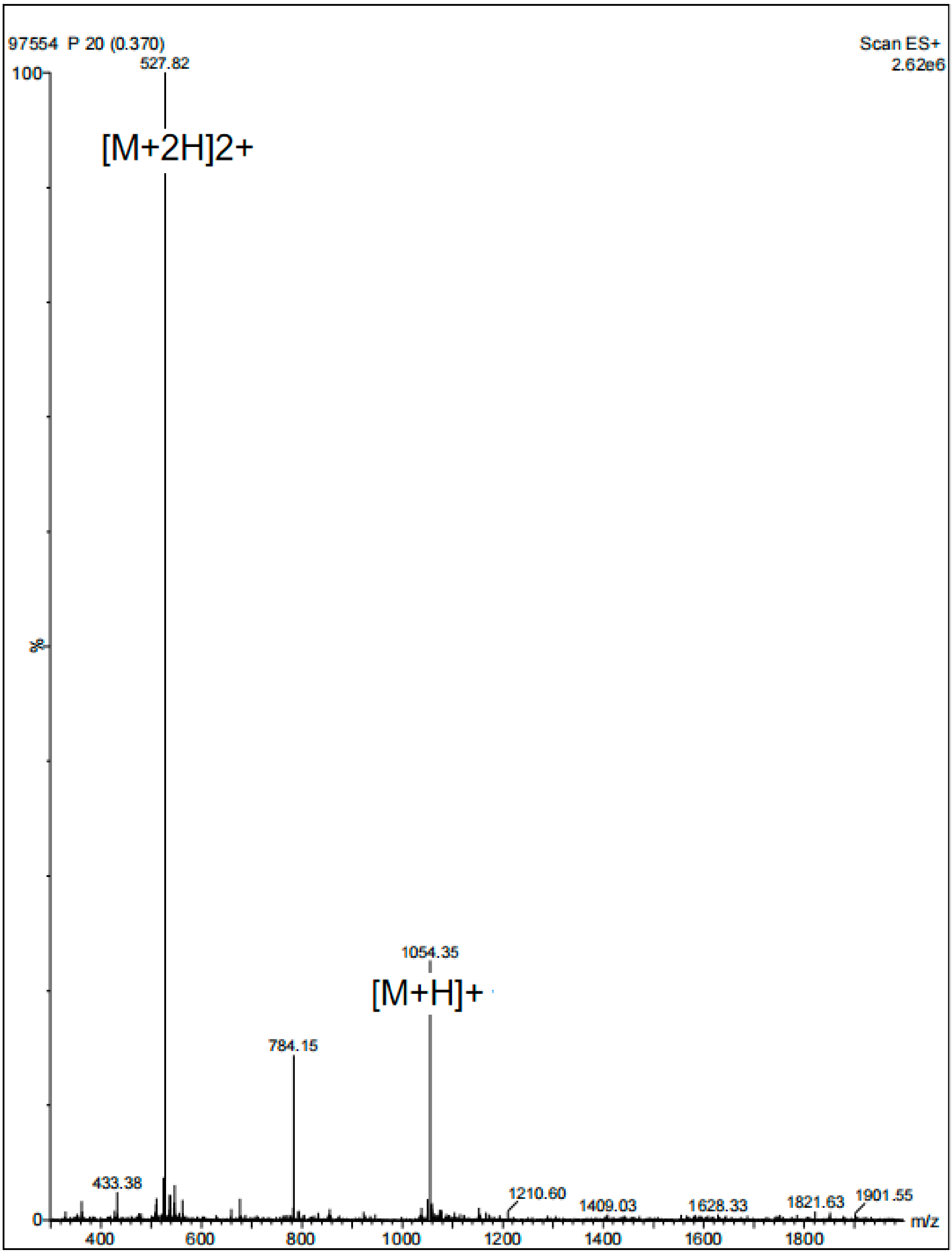

Supplement: Supplementary file 1 [file molecules-30-04818-s001.zip › molecules-4017863-supplementary.pdf]
